# Canine Leishmaniosis in Greece: An Updated Countrywide Serological Study and Associated Risk Factors

**DOI:** 10.3390/pathogens10091129

**Published:** 2021-09-02

**Authors:** Isaia Symeonidou, Athanasios Angelou, Alexandros Theodoridis, Georgios Sioutas, Elias Papadopoulos

**Affiliations:** 1 Laboratory of Parasitology and Parasitic Diseases, Faculty of Health Sciences, School of Veterinary Medicine, Aristotle University of Thessaloniki, 54124 Thessaloniki, Greece; isaia@vet.auth.gr (I.S.); amangelo@vet.auth.gr (A.A.); gsioutas@vet.auth.gr (G.S.); 2 Laboratory of Animal Production Economics, Faculty of Health Sciences, School of Veterinary Medicine, Aristotle University, 54124 Thessaloniki, Greece; alextheod@vet.auth.gr

**Keywords:** Canine leishmaniosis, serology, risk factors, Speed Leish K^®^, Greece

## Abstract

Canine leishmaniosis (*Leishmania infantum*) is a zoonotic disease that affects dogs worldwide. Greece is enzootic for this disease, and updated data for its current distribution are of major importance. The aim of this cross-sectional serological study was primarily to update the current knowledge of *Leishmania infantum* seropositivity status within the asymptomatic Greek canine population and, furthermore, to assess the possible climatological and other risk factors. In total, sera of 1265 asymptomatic dogs were collected from all prefectures of the country. A questionnaire that included all individual dog information was completed for all animals. The Speed Leish K^®^ canine *Leishmania* antibody test kit (BVT Groupe Virbac, France) was employed. Potential risk factors were evaluated utilizing logistic regression models. Overall, 13.8% (*n* = 175) of the sampled dogs were seropositive to *Leishmania infantum* originating from all geographical departments of the country, whereas most prefectures had at least one seropositive animal. Outdoor living, high mean humidity, low mean wind speed and high total annual rainfall were found to increase the seropositivity status against the parasite. Conclusively, *Leishmania infantum* remains a common parasite challenge in the asymptomatic canine population of Greece, and therefore, its early diagnosis and effective prevention are significant in the country.

## 1. Introduction

Canine leishmaniosis (CanL), caused by the protozoon parasite *Leishmania infantum* (*L. infantum*, syn. *L. chagasi*), is a vector-borne disease that can be life-threatening [1,2]. Dogs are the primary peridomestic reservoir hosts for this parasite [3,4], which can also infect humans through bites of sandflies of the genus *Phlebotomus* [5], causing Human Visceral Leishmaniasis (HVL), a disease with high morbidity and mortality rates [2]. 

CanL remains a challenge for veterinary practitioners in terms of diagnosis and control [1]. The determination of infected dogs is complex due to various reasons. Dogs that acquire the infection may remain asymptomatic for months or even for years and, in some cases, for their entire lives [6,7]. Indeed, previous canine population studies in enzootic areas have revealed that only a proportion of infected dogs manifest the clinical disease, while another fraction develops a subclinical infection, and yet another percentage of animals, which are resistant to this protozoon, manage to resolve the infection and self-heal [8,9,10]. Additionally, the incubation period before the manifestation of symptoms may last from months to years [6]. 

Moreover, symptomatic dogs exhibit a wide spectrum of nonspecific clinical signs ranging from mild local skin lesions to fatal systemic syndromes [7,10,11] due to the various pathogenic pathways of the disease and the diverse induced immune responses of the hosts [12]. It should be stressed that experimental studies have demonstrated a correlation between the infectivity of sandflies and antibody response [3] and have confirmed that asymptomatically infected dogs can transmit *L. infantum* and establish infections in the phlebotomine vectors [3,13,14]. Therefore, the early and accurate detection of carriers is essential from a clinical and epizootiological point of view. 

Serology is a practical diagnostic approach for confirming the disease and identifying infected animals [7]. Commercial immunochromatographic test kits that detect circulating anti-*L. infantum* antibodies are preferred in extended epizootiological surveys of CanL, since they are labour-saving, of affordable costs and can be performed rapidly on-site [15]. Regardless of their limitations, such assays can detect the majority of symptomatic and a significant proportion of asymptomatic dogs [9,16,17]. Mediterranean countries are enzootic [18], and seropositivity has been reported as high as 54.1% [19] and 57.1% of the canine population in some areas [20]. In Greece, several previous surveys, regional or cross-sectional, have been carried out, but current data are lacking. These surveys used different diagnostic approaches, but none of them employed up to now the Speed Leish K^®^ canine *Leishmania* antibody test kit (BVT Groupe Virbac, France) [21,22,23,24,25]. Noteworthy, Sifaki-Pistola et al. [25] developed a prediction model, employing a spatial analysis based on the available epizootiological data [24] and a specially developed questionnaire, according to which areas of the country are expected to have dog seropositivity levels of 3-55%, referring to both asymptomatic and clinically sick dogs.

The objective of this study was to perform an updated countrywide sero-epizootiological study within the asymptomatic canine population regarding the spread of *L. infantum* in Greece by the detection of specific antibodies against *L. infantum*. The aim of the study was to investigate the serological status of these dogs, keeping in mind that this status is only a correlate of CanL. In addition, this study aimed to investigate in depth the possible interacting risk factors enhancing this infection. 

## 2. Results

Gender was almost evenly distributed within the studied population, with 573 (45.2%) male and 692 (54.8%) female dogs. The majority of the animals (*n* = 724, 58.3%) were over three years old, whilst 541 out of 1265 dogs (42.7%) were up to three years old. The age ranged from 1 to 8 years of age (mean age approximately 3 years). Most of the studied animals were crossbreed dogs (*n* = 648, 51.2%), while the rest belonged to various purebred canine breeds, i.e., 79 (6.2%) were English Setters and 49 (3.8%) German Shepherds, 45 (3.5%) Greek Greyhounds, 41 (3.2%) Dalmatians, 39 (3%) Golden Retrievers, 38 (3%) Greek Kokoni and 35 (2.7%) Rottweilers. The rest were breeds with a very small number of sampled dogs to report. Most dogs lived outdoors (*n* = 912, 72%), while only 353 (18%) lived indoors and spent some daily time outside during walking with their owners. The mean values for the environmental parameters were the following: 15.9 °C, −5.5 °C and 38.3 °C for the mean, minimum and maximum temperatures, respectively; 69.7% for the mean humidity, 10.7 knots for the mean wind speed and 554 mm for the total annual rainfall.

**Figure 1:**
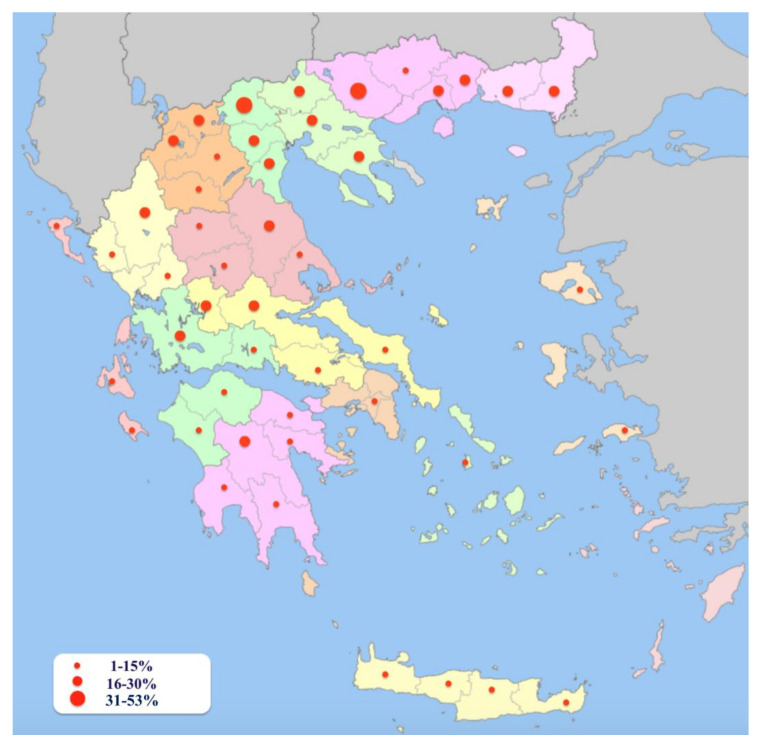
Distribution of *Leishmania infantum* seropositive dogs and the recorded seropositivity rates across the country.

Overall, 13.8% (*n* = 175) of the sampled dogs were seropositive to *L. infantum* (95% confidence interval (CI): 12.0%–15.8%). In detail, seropositive dogs were found in all geographical departments of the country, and most prefectures had at least one seropositive animal. Macedonia and Thrace were the geographical departments of the country with the highest rates of seropositive animals, i.e., 26% (95% CI: 20.5–26.0) and 20% (95% CI: 14.0–26.0), respectively, while the lowest rates were detected in the Aegean and Ionian Islands (5.1%, 95% CI: 2.0–8.2 and 7.1%, 95% CI: 2.0–12.3, respectively). The seropositivity rate per prefecture ranged from 0% to 53%. The distribution of seropositive dogs and the recorded seropositivity rates across the country are illustrated in Figure 1. In detail, Table 1 summarizes the seropositivity status of the examined animals per geographical department and prefecture. 

In the logistic regression, 12 risk factors (predictors) were used to investigate their association with the *L. infantum* seropositivity status. The factors that estimated to have a univariable association with the *L. infantum* status at a significance level of 0.10 (*p*-value ≤ 0.10) are presented in Table 2. The results indicate that five out of the 12 predictors were strongly related to *L. infantum* seropositivity in dogs. Subsequently, these five factors were incorporated into the final multivariable logistic regression analysis using a backward-stepwise selection approach. 

Four factors were significantly associated with the *L. infantum* status in the final multivariate model (Table 3). According to the estimations based on the results of the multivariate logistic regression, dogs that live indoors with low levels of humidity have a predicted probability of being positive for *L. infantum* of 7.9%. This probability is increased to 13.6% when the level of humidity is high, indicating the great impact of this factor on the disease. The probability of being positive increases to 24.6% when the dogs live outdoors with high levels of humidity and the absence of strong winds. Moreover, dogs that live outdoors with high levels of humidity and rainfall have a predicted probability of being positive for *L. infantum* of 14.8%. The probability for the dogs that live outdoors in high rainfall areas is 8.9%, while for those that live outdoors in strong winds areas, the probability is only 1.8%. Finally, the probability of being positive for *L. infantum* is decreased to 0.8% when the dogs are kept indoors where the level of humidity and rainfall are high.

## 3. Discussion

CanL represents a disease of major veterinary significance with zoonotic potential [1,2]. Many surveys have concluded that this infection is widely distributed in the Mediterranean basin with remarkable variability in seroprevalence data, depending on the region and ranging between 4.7% and 57.1%, both percentages reported in different regions of Spain [20,26]. In more detail, in the neighboring Italy, the median 30 years prevalence calculated from 377 canine serosurveys performed in the country’s south was 18.0% [27]. Dog seropositivity is reported to have reached up to 7.1% in Northeast Italy [28], 17.9% in the South Campania region [29], 34.6% in the Aeolian islands of Sicily [30] and a striking 54.1% on Lampedusa Island [19]. Likewise, in the Iberian Peninsula, extensive surveys recorded seropositive dogs from 5.4% up to 8.1% in the Madrid region [26,31,32], 10.2% in Northeast Catalonia [33], 19.5% in Girona Province [34], 26.0% on the Island of Mallorca [8], 35.6% in some regions of Northern Spain [35] and 57.1% in the Balearic Islands, which is the highest seroprevalence documented in Europe [20]. A high seroprevalence (56.0%) has also been recorded in the Municipality Vila de Rei in Central Portugal [36], while in the northern part of the country, it was 18.7% [37]. Previous studies in neighboring countries of Greece, such as in Albania and Kosovo [38,39], as well as in Turkey and Northern Cyprus [40], employing both serological and molecular diagnostic tools confirmed the presence of CanL within the local canine population, thus denoting that *L. infantum* is also distributed in the broader region of Eastern Europe, including the Balkan Peninsula [41]. 

In Greece, several previous studies included asymptomatic dogs and reported different seroprevalences of CanL from 12.3% [21] up to 24.4% [22]. As expected, when both asymptomatic and symptomatic animals were included, the prevalence estimate reached up to 50.2% [24]. It should be pointed out that the design of each survey varies regarding inclusion criteria, serological method, sample size, time of sampling and other factors, and all of these can introduce significant variations in the seroprevalence estimates. Therefore, comparing any results to these of other studies must be performed with caution [23,27,34]. In the present study, the serological screening of a substantial and representative canine population with samples deriving from each prefecture of the country was conducted. The results of this multicenter study presented updated data on the epizootiology of *L. infantum* in asymptomatic Greek dogs. The overall prevalence of positive animals to *L. infantum*-specific antibodies was 13.8%, and remarkably, in all the geographical departments, seropositive dogs were detected. The above confirms that Greece remains a highly enzootic country for CanL, as previously suggested by the aforementioned surveys.

Although, in all the geographical departments seropositive dogs were detected, the prevalence rate per prefecture ranged from 0% to 53%. It should be noted that the number of sampled animals differed in various areas. As depicted in the detailed epizootiological map, the distribution of seropositive dogs across the north–south and east–west axes of the country varies considerably (Figure 1). This manner of distribution has been confirmed in a previous survey in Greece, where the prevalence estimates ranged from 6.5% up to 50.2%, depending on the prefecture [24]. The display of different epizootiological profiles of CanL throughout a country is expected, as noted in other enzootic areas [20]. This pattern is attributed to a plethora of factors, such as the diverse geographical and climatic or socioeconomic conditions of different areas, as well as the presence of canine and vector populations [42]. 

Although asymptomatic seropositive dogs are less infectious to phlebotomine vectors than their symptomatic counterparts [43], they still can transmit the parasite and favor its unnoticed spread among dogs [1,3,14]. Consequently, the early detection of the proportion of these permanent reservoirs of *L. infantum*, which seems to be relatively high in enzootic regions, is a crucial point for the control of CanL, regardless of if they develop symptoms or not. This also applies in non-enzootic countries, as it is stated that the infection spreads northward in Europe through dog movement [18]. Moreover, considering that CanL is relevant to public health, estimating the prevalence rate within the canine population constitutes a very effective method for assessing the risk of infection for susceptible humans, i.e., immunosuppressed individuals. This monitoring of the disease is of particular interest in urban and semi-urban areas where humans and dogs live in close vicinity and can raise awareness among public health practitioners towards implementing more efficient protective measures so as minimize or even eliminate the risk of human infection [44,45,46]. On top of the above, CanL imported cases exceeded 700 in the last few years in traditionally non-enzootic countries in Europe [18]. Therefore, given that Greece is a very popular touristic destination, which annually attracts millions of people accompanied by their pets, identifying high-risk areas is of great importance. Such knowledge would assist towards targeted effective prevention efforts for traveling pets. Moreover, the legislation regarding traveling pets should include thorough testing for CanL. 

Serological assays could be sensitive, specific and proven efficient for estimating the distribution of CanL [16,46]. Seropositivity, in most cases, is well-correlated with the clinical manifestations of the disease, as low antibody titers are usually detected in infected dogs that have not developed the clinical disease, while increasing antibody levels commonly develop in persistent symptomatic infections [1,47]. These diagnostic tools are ideal for carrying out sero-epizootiological mass-screening surveys and fast routine diagnostic practice [9,17]. 

However, a diagnosis mediated by serology has limitations and often underestimates the number of *L. infantum* infected dogs in enzootic areas [9]. It has been widely accepted that serological screening identifies most of the symptomatic dogs [11,48] and a proportion of the asymptomatic ones, but it fails to identify infected dogs in the absence of specific antibodies, i.e., during the prepatent period, before seroconversion [49,50], as well as the animals that will never seroconvert [50,51] and, also, those that were seropositive and reverted seronegative while remaining parasite positive [51,52]. For providing an accurate estimation of the actual infection rate, the employment of advanced diagnostic tools such as molecular and immunoblotting techniques [6,8] or cell-mediated assays [47] is required. Indeed, a study conducted in Greece using Polymerase Chain Reaction (PCR) and Indirect Immunofluorescence Assay (IFAT) tested 73 apparently healthy hunting dogs and recorded prevalence estimates of 63% and 12.2%, respectively, indicating that the occurrence of infection is actually much higher than that recorded with the serological investigation [21]. To be concise, the estimates reported in seroprevalence surveys are expected to be between the prevalence of the disease and the infection rate [1,6,8]. 

The Speed Leish K^®^ (BVT Groupe Virbac, La Seyne sur Mer, France) is a commercially available rapid assay based on the immunochromatographic detection of specific anti-*L. infantum* kinesin antibodies. This diagnostic tool uses as a capture antigen a complex of recombinant kinesins and displays a sensitivity of 96.3% and a specificity of 100% compared to IFAT and immunoblotting [46]. Noteworthy, the Speed Leish K^®^ is suitable for the diagnosis of leishmaniosis in vaccinated animals, as it has been demonstrated that it does not detect antibodies induced by vaccination, such as those provoked with LiESP/QA-21, a vaccine made of purified proteins from *L. infantum* [53]. Therefore, a positive result marked by the presence of antibodies indicates contact with the parasite. Regarding the possible serological cross-reactivity with *Trypanosoma* species that could result in false-positive samples [1], it should be mentioned that, in Greece, trypanosomes do not infect dogs and that infections by other *Leishmania* species, except *L. infantum,* are rare [15,23,54]. 

Another objective of the present study was to determine the CanL-associated risk factors. Several studies conducted over the last years have identified certain risk factors for the occurrence of CanL. These factors are related either to the host or the environmental conditions. 

Regarding the factors that predispose the host to infection, gender was not associated with canine seropositivity for *L. infantum* in Greece. Contrary to our study, other research groups [7,55,56,57] have revealed a significantly higher seroprevalence in male dogs compared to female ones. This finding has been attributed to the immunomodulating properties of testosterone in dogs that result in different host immune responses between genders and, therefore, influence the resistance or susceptibility to infection [58], as well as to the fact that male dogs roam more than females [42].

The risk factors analysis in the present study did not associate age with the infection rate, corroborating the results of França-Silva et al. [59], who observed that, regardless of age, animals had the same probability of acquiring the infection. However, according to the literature, several authors claim that age seems to be an important risk factor for CanL [10,34,35,60,61]. The higher frequency of seropositivity among older dogs can be explained due to the fact that adult dogs usually remain outside for extended time periods and, thus, have an increased risk of exposure to sandflies [34,35] or due to concomitant diseases that weaken the immune system [55]. Furthermore, it has been attributed to the long serological latency of CanL [6], which implies that seropositivity persists for prolonged periods of time. The chronicity of the infection has an accumulative effect in older patients and seroprevalence increases as dogs age [61].

The effect of dog breeds was absent in this study, whereas some previous surveys identified the dog breed as a variable associated with seropositivity. Some researchers support the hypothesis that purebred dogs have a higher risk of acquiring CanL compared to crossbreed dogs [57]. In this regard, it has been indicated that certain breeds such as the German Shepherd, Boxer, Rottweiler and Cocker Spaniel tend to be more susceptible to the development of the disease [10,55,59], while crossbreed and autochthonous dogs appear more resilient to the parasite [9,62]. Opposed to the above, some authors claim that crossbreed dogs have a higher risk of infection than purebred dogs. Crossbreeds often remain loose in the vicinity and without adequate management. It is common that crossbreed dogs are removed from the streets, and even though they receive shelter and food, they remain without proper care by their owners, unlike dogs with a defined breed [34,42,56,63]. 

In this study, the risk of infection was higher in dogs kept mainly outdoors than those with an indoor lifestyle. The dog’s lifestyle is a controversial factor, with several authors proposing it is not relevant whether animals live mainly indoors or outdoors [23,49,58,64] and others who affirm that seropositivity is undoubtedly associated with an outdoor lifestyle, verifying the results of the present study [32,57,65,66]. Logically, the spread of the infection is expected to be higher in dogs spending most of the time outdoors than those living indoors, because the latter are more unprotected from vector action. Indeed, research in enzootic areas has suggested that animals sleeping outdoors can be stung by hundreds of sandflies, and it has been speculated that this cumulative exposure favors seroconversion, since the parasite is repeatedly inoculated into these dogs [7]. 

Among the examined risk factors that are related to the environmental conditions, the high mean humidity (>69.7%), low mean wind speed (≤ 10.7 knots) and high total annual rainfall (>544 mm) were found to increase the seropositivity status against *L. infantum*. To this regard, it is well-established that the risk of CanL in enzootic areas depends on the sandfly species density [67,68]. Weather variables such as temperature, humidity, winds and rainfall have a critical influence on the distribution, activity and survival of vectors and, thus, render leishmaniosis as a climate-sensitive parasitosis [69,70,71].

Our findings supported a univariable association with *L. infantum* seroposivity status for a regional mean temperature of ≤15.9 °C, but this factor was not significantly associated with the final multivariate model (Table 3). It is apparent that, most likely, various interacting factors are driving these results. For example, in cooler areas, due to the shorter sandfly activity period, a more aggressive feeding may occur, thus leading to a higher transmission potential. Maybe other factors such as non-vectorial transmission routes (i.e., venereal, vertical, dog-to-dog transmission or blood transfusion) could be involved, as they occur in non-endemic areas. Nevertheless, the possibility that these findings are random cannot be excluded. It should be noted that sandflies are ectotherms, and their distribution and activity depend on the temperature, with an average temperature between 20–35 °C considered optimal [72]. The high mean environmental temperature has been reported as an important risk factor for dog seropositivity in surveys conducted in various countries [27,73,74] and in Greece [24]. 

A climate variable that has been significantly associated in the final multivariate model of this study with a higher risk of CanL seropositivity is the high mean humidity. In detail, a mean humidity of >69.7% has been positively associated with seropositivity. According to Santos et al. [75], high humidity provides optimal conditions for the phlebotomine sandfly species, which disseminate the disease. In detail, high relative humidity is a prerequisite for the development of the sandfly larvae, and adult *Phlebotomus* spp. are very sensitive to desiccation [76].

A low wind speed is a climatic condition commonly observed to favor the presence of phlebotomine species and, consequently the spread of infection. *Phlebotomus* spp. are poor fliers, and the wind generates drafts preventing them from entering buildings and discouraging their activity [77]. In Central Portugal, the highest sandfly density has been recorded in the absence of strong winds [74]. Sandfly activity in Greece is usually from April–October [68]. During this period, the winds blowing over most parts of Greece are steady from the northern to northeastern/northwestern directions and scarcely reach gale force strength [78]. Data from the current study reinforces this hypothesis, since a low mean wind speed, i.e., a wind speed of ≤ 10.7 knots, increased the seropositivity status against *L. infantum*.

A total annual rainfall that exceeds 544 mm was identified as an environmental parameter related to an increased risk of a seropositive status. In the same frame, a greater frequency and diversity of sandflies were noted in the rainy season (87.9%) than in the dry season (12.1%) in Brazil [79]. Higher precipitation may directly increase sandfly populations [80]. However, rainfall has been negatively associated with sandfly activity in other Mediterranean regions [81,82,83]. 

In our study, we failed to demonstrate any effect of altitude on the occurrence of CanL. Sandflies can be found over a broad altitudinal range [68]. The altitude may act on sandfly distribution through a variety of habitats and a gradient change of the temperature (−0.6 °C per 100 m) [84]. In this frame, a survey conducted in the Greek Islands has reported an impact of altitude upon *Phlebotomus* spp. fauna distribution [72]. Another study carried out in the country recorded the majority of the infected animals in low altitudes (mean altitudes of 171 and 151 masl) [24]. Correspondingly, Giannakopoulos et al. [84] noted that the highest percentage of high-risk areas for CanL in Greece was located at low altitudes (<200 masl).

## 4. Materials and Methods

### 4.1. Study Area, Dogs and Collection of Serum Samples

Overall, 66 locations were chosen from all 54 prefectures of Greece evenly distributed along the country’s north-south and east-west axes. A total of 1265 dogs were sampled for one year (2020), including 238 (18.8%) from Macedonia, 196 (15.5%) from the Aegean Islands, 189 (15.0%) from Central Greece, 170 (13.4%) from Thrace, 142 (11.2%) from Peloponnese, 98 (7.7%) from the Ionian Islands, 90 (7.1%) from Thessaly, 73 (5.8%) from Crete and 69 (5.5%) from Epirus (Table 1). The number of sampled dogs was as high as possible given the practical limitations. All the dogs included in the study were autochthonous, with no history of travelling abroad, had a normal physical examination and were older than 6 months. The samples were collected from each dog during routine visits at veterinary practices after receiving the owner’s informed consent for every animal. A blood sample was collected mainly from the cephalic or jugular vein into vacutainer tubes without an anticoagulant and stored at 4 °C for a maximum of 24 h before centrifugation at 2200 rpm for 20 min; after which, the serum was separated from the clot. The serum samples were then stored at 4 °C until further examination. A detailed questionnaire that included all individual information, such as gender, age, breed and living conditions (outdoors or indoors), was filled in for each sampled dog. 

### 4.2. Serological Analysis

The serum samples were examined in the Laboratory of Parasitology and Parasitic Diseases of the Aristotle University of Thessaloniki by the Speed Leish K^®^ (BVT Groupe Virbac, La Seyne sur Mer, France). This test comprises a dipstick device, which is based on the immunochromatographic detection of specific anti-*L. infantum* kinesin antibodies. The kits were stored at room temperature and were used according to the manufacturer’s instructions. The results were read twice; firstly, after 15 min, and thereafter, a reading was performed at 30 min, as suggested by the manufacturer.

### 4.3. Climatological and Altitude Data

Climate data were collected for each of the examined regions, including the mean, minimum and maximum environmental temperatures (°C); mean humidity (%); mean wind speed (knots) and total annual rainfall (mm). The Hellenic National Meteorological Services (HNMS) and the "Meteo View" portal provided the meteorological data used in the current study. The HNMS is the official government agency that provides the national weather forecast, whereas the "Meteo View" is a web platform based on the Geographic Information System (GIS) tool. For each examined region, the nearest meteorological station was chosen, and the mean values for each metric were acquired for further analyses.

The regional altitude data used in the current study were retrieved from a Digital Elevation Model (DEM) and NASA’s Shuttle Radar Topography Mission (SRTM), with an accuracy rate of approximately ± 1.73m, which is considered sufficient for such surveys [85]. The altitude readings were collected from points on the map corresponding to the exact sampling location. Three altitude zones were considered: from 0 to 100, from 101 to 400 and from 401 to 900 meters above the sea level (masl).

### 4.4. Data Handling and Statistical Analyses

The seroprevalence was determined as the proportion of positive dogs to the total number of studied animals. Univariate binary logistic regression models were applied to assess the effects of possible risk factors, either those related to the host such as gender, age, breed and living conditions (outdoors or indoors) or those related to environmental parameters, such as the temperature (mean, minimum and maximum); mean humidity; mean wind speed; total annual rainfall and altitude, on the probability of seropositive status for *L. infantum*. The data were analyzed using IBM SPSS Statistics 25.0. Univariable associations with a *p*-value ≤ 0.10 were selected for inclusion in the multivariate logistic regression model. A backward multivariable logistic regression model was applied using as predictors the variables that presented a strong univariable association with the *L. infantum* seropositivity status, with a *p*-value >0.05 as the criterion for a variable removal. Hosmer-Lemeshow goodness of fit was estimated for the assessment of the multivariable logistic regression model. 

## 5. Conclusions

In conclusion, the present study evidenced that CanL remains enzootic in Greece. Besides the risks for dogs and humans, updating the epizootiological knowledge on CanL throughout Greece is of importance due to the great touristic appeal of the country. Such information on disease surveillance can help define targeted interventions to reduce CanL and evaluate their effectiveness. The presence of antileishmanial antibodies in serum indicates exposure to the parasite; consequently, follow-up studies are required to correlate between seropositivity and the prospect of developing a persistent infection. Our findings highlight that a dog’s habitat, high mean humidity, low mean wind speed and high total annual rainfall are positively correlated with the seropositivity status against the parasite. To fill in the gaps, there is a necessity for additional studies regarding the in-depth identification of the risk factors and high-risk areas, as well as large-scale entomological surveys, especially in view of climate change, in the mainland and islands of Greece.

## Figures and Tables

**Table 1:** Seropositivity status of 1265 sampled dogs examined for specific antibodies against *L. infantum* per geographical department and prefecture in Greece.

Geographical Department	Prefecture	Sampled Animals	Seropositive Animals (%, 95% CI)
	Evros	80	14(17.5)
**Thrace**	Rodopi	20	5(25)
	Xanthi	70	15(21.5)
		170	34 (20.0, 14.0–26.0)
	Kavala	25	7(28)
	Drama	20	3(15)
	Serres	15	8(53)
	Chalkidiki	20	6(30)
	Thessaloniki	30	6(20)
	Kilkis	20	5(25)
**Macedonia**	Pella	13	6(46)
	Florina	30	5(16)
	Imathia	25	5(20)
	Pieria	20	3(15)
	Grevena	20	3(15)
	Kozani	20	2(10)
	Kastoria	20	3(15)
		238	62 (26.0, 20.5–31.6)
	Ioannina	12	3(25)
	Thesprotia	20	2(10)
**Epirus**	Preveza	20	0(0)
	Arta	17	2(11.7)
		69	7 (10.1, 3.0–17.3)
	Larissa	30	5(16)
	Magnisia	20	2(10)
**Thessaly**	Karditsa	20	3(15)
	Trikala	20	1(5)
		90	11 (12.2, 5.4–19.0)
	Attica	97	10(10.3)
	Euboea	19	1(5.2)
	Viotia	21	3(14.2)
**Central Greece**	Fokis	20	1(5)
	Fthiotis	8	2(25)
	Evrytania	14	4(28.5)
	Aetolia-Acarnania	10	2(20)
		189	23 (12.2, 7.5–16.8)
	Corinth	20	2(10)
	Achaea	20	1(5)
	Ilia	21	1(4.7)
**Peloponnese**	Arcadia	16	3(18.7)
	Argolida	15	2(13)
	Messinia	30	4(13.3)
	Lakonia	20	1(5)
		142	14 (9.8, 4.9–14.8)
	Chios	14	0(0)
	Mytilene	30	4(13.3)
**Aegean Islands**	Samos	25	2(8)
	Cyclades	102	4(3.9)
	Dodecanese	25	0(0)
		196	10 (5.1, 2.0–8.2)
	Corfu	38	5(13.1)
**Ionian Islands**	Kefallinia	20	1(5)
	Lefkada	20	0(0)
	Zante	20	1(5)
		98	7 (7.1, 2.0–12.3)
	Chania	18	2(11)
	Heraklion	20	2(10)
**Crete**	Rethymno	15	2(13.3)
	Lasithi	20	1(5)
		73	7 (9.6, 2.8–16.4)
**Total**		1265	175 (13.8, 12.0–15.8)

**Table 2:** Risk factors associated (*p*-value ≤ 0.10) with the *L. infantum* status using univariable logistic regression.

Description of Factors	Odds Ratio	95% CI		*p*
Habitat indoors	0.48	0.29	0.78	0.004
Mean Temperature ≥15.9 °C	0.56	0.39	0.81	0.002
Mean Humidity ≥69.7%	1.55	1.12	2.14	0.008
Mean Wind Speed ≥10.7 knots	0.14	0.04	0.43	0.001
Total Annual Rainfall ≥554 mm	0.69	0.48	0.99	0.047

**Table 3:** Risk factors associated (*p*-value ≤ 0.05) with *L. infantum* status using a multivariable logistic regression model.

Description of Factors	b ^1^	SE(b) ^2^	*p*	Odds Ratio	95% CΙ	
Intercept	−1.728	0.131	<0.001			
Habitat- indoors	−0.728	0.257	0.005	0.48	0.29	0.80
Mean Humidity ≥69.7%	0.609	0.176	0.001	1.83	1.30	2.59
Mean Wind Speed ≥10.7 knots	−2.265	0.594	<0.001	0.10	0.03	0.33
Total Annual Rainfall ≥554 mm	−0.625	0.196	0.001	0.54	0.36	0.79

^1^ b: Regression coefficient, ^2^ SE(b): standard error of b, Hosmer–Lemeshow goodness-of-fit test, *p*-value = 0.8083.

## Abbreviations

CanL: Canine Leishmaniosis; *L. infantum*: *Leishmania infantum*; HVL: Human Visceral Leishmaniosis; HNMS: Hellenic National Meteorological Services; GIS: Geographic Information System; DEM: Digital Elevation Model; SRTM: Shuttle Radar Topography Mission; masl: meters above sea level; CI: Confidence Interval; PCR: Polymerase Chain Reaction; IFAT: Indirect Immunofluorescence Assay.
